# Topophilia and the Quality of Life

**DOI:** 10.1289/ehp.7467

**Published:** 2004-11-22

**Authors:** Oladele A. Ogunseitan

**Affiliations:** Department of Environmental Health, Science, and Policy, University of California, Irvine, California, USA

**Keywords:** ecosystems, mental health, nature, quality of life, restorative environments, stress, topophilia

## Abstract

With this research I tested the hypothesis that individual preferences for specific ecosystem components and restorative environments are significantly associated with quality of life (QOL). A total of 379 human subjects responded to a structured 18-item questionnaire on topophilia and to the 26-item World Health Organization’s Quality of Life (WHOQOL-Bref) instrument. Confirmatory factor analyses revealed four domains of topophilia (ecodiversity, synesthetic tendency, cognitive challenge, and familiarity) and four domains of QOL (physical, psychological, social, and environmental). Synesthetic tendency was the strongest domain of topophilia, whereas the psychological aspect of QOL was the strongest. Structural equation modeling was used to explore the adequacy of a theoretical model linking topophilia and QOL. The model fit the data extremely well: χ^2^ = 5.02, *p* = 0.414; correlation = 0.12 (*p* = 0.047). All four domains of topophilia were significantly correlated with the level of restoration experienced by respondents at their current domicile [for cognitive challenge: *r* = 0.19; *p* < 0.01; familiarity: *r* = 0.12; *p* < 0.05; synesthetic tendency: *r* = 0.18; *p* < 0.01; ecodiversity (the highest value): *r* = 0.28; *p* < 0.01]. Within ecodiversity, preferences for water and flowers were associated with high overall QOL (*r* = 0.162 and 0.105, respectively; *p* < 0.01 and 0.05, respectively). Within the familiarity domain, identifiability was associated with the environmental domain of QOL (*r* = 0.115; *p* < 0.05), but not with overall QOL. These results provide a new methodologic framework for linking environmental quality and human health and for implementing evidence-based provision of restorative environments through targeted design of built environments to enhance human QOL.

Mental stress is an underestimated and growing component of disease burden in many parts of the world (Saxena et al. 2003). The roles of both natural and constructed environments in relieving mental stress have long been suspected but are poorly understood. Tuan (1999) originally defined topophilia as the affective bond between people and place or environmental setting. Topophilia is presumed to be a vivid and personal experience, but research is scarce on the determinants of individual preferences and on the potential health benefits derived from such experiences. The few existing studies have not adequately deconstructed the confounding of affective and cognitive processes in aesthetic response versus tangible health outcomes (e.g., Parsons 1991; Ulrich 1979). Furthermore, quantitative assessments of the values associated with the considerable financial investment by societies in naturalistic environmental design, landscape architecture, and ecosystem conservation through wildland natural preserves are rare. When available, the results of such studies are often inconclusive or contradictory. The proximate causes of particular topophilia are embedded in measurable characteristics: environmental perception, defined as the response of senses to external stimuli and purposeful activity; attitude, or ingrained cultural stances; and values, the rank-ordered conception of preferences that emerge following a personalized exercise in trade-offs among alternative scenarios. Environmental designers have long exploited the basic ideas of topophilia to create presumably attractive surroundings that restore mental health based on the use of materials, sensory stimuli, and arrangements that remind people of the place and environmental settings that are comforting and/or associated with healing potential (Carlson 2000; Porteous 1996).

In the tradition of environmental psychology, “restorative environments” are defined as specific geographical contexts that renew diminished functional capabilities and enhance coping strategies and resources for managing stress (Hartig and Staats 2003). There is also general consensus that measuring restoration according to this definition is complicated. In urban cultures where restorative environments are conventionally linked to few and remote vestiges of forest wilderness or pristine water views, it is increasingly important to understand the role of landscape design and public art in providing sanctuaries where a sense of balance can be restored to hectic lifestyles. However, parameters such as age, sex, ethnic background, and socioeconomic status have powerful influences on individual and group perception of restorative environments as defined by artificial public spaces in confined urban centers (Hartig et al. 2003; Laumann et al. 2003). In this research project I sought to identify common features of preferred restorative environments in a sample population according to the categories typically associated with topophilia: synesthetic tendency (commingling of sensory stimuli and the memory of place), environmental familiarity, cognitive challenge, and ecodiversity (Janzen 1998; Tuan 1993, 1999). The specification of topophilic preferences is potentially more informative if the preferences are linked to tangible benefits for human health and welfare. In this regard, the literature on restorative environments has lacked a quantitative measure of restoration, although there have been some preliminary empirical excursions into the putative linkages between individual environment preferences and restoration (Staats et al. 1997, 2003; van den Berg et al. 2003). Those studies typically request that stressed subjects declare their preference (e.g., which is more “beautiful”?) for either a forested landscape or the concrete world of an urban downtown. Hence, the fine-level characteristics of built versus natural ecosystems have not been adequately captured (Ulrich 1993).

In the present study, I assessed the mental health profile of respondents to the topophilia survey by means of the World Health Organization’s quality of life survey instrument (WHOQOL-Bref). The WHO defines quality of life (QOL) as “an individual’s perception of their position in life in the context of the culture and value systems in which they live, and in relation to their goals, expectations, standards and concerns” (WHOQOL Group 1994, 1995, 1998a, 1998b). Both the 100-question (WHOQOL-100) and 26-question (WHOQOL-Bref) versions have been validated across many cultures, in several countries, and for different contexts of health, well-being, and occupational stress (Kuyken et al. 1994; Nasermoaddeli et al. 2003; Skevington et al. 2004; Weatherall et al. 2004). WHOQOL-Bref is recommended when it is advisable to minimize the time burden on respondents. Furthermore, it has been shown to have excellent psychometric properties of reliability, and it performed well in tests of validity across the four domains of health, namely, physical health, psychological well-being, social relationships, and environmental support (Saxena et al. 2001).

As a seminal exploration of the linkages between QOL and preferred environmental and ecosystem features, the present study explicitly posed the hypothesis that those exhibiting high QOL are more likely to describe their domicile as providing access to restorative environments defined by specific components of the landscape. I further hypothesized that preference for the specific topophilia domain of ecodiversity is associated with high QOL. The hypotheses were tested by statistical analyses of responses to structured questionnaires. The population sampled for this study identified preferences for water bodies, flowers, and spatial familiarity restoration.

The results provide insight into specific aspects of ecosystems and artificial landscapes that are more likely to support restoration and the enhancement of QOL. Importantly, the set of methods developed here provides a strategy for future investigations addressing the response of diverse populations in different urban environments to various aspects of natural and artificial topography.

## Materials and Methods

### Human subject pool.

Human subjects for the study were recruited at the University of California at Irvine between August 2001 and August 2002. For most respondents, the campus represented both residential and work environments during periods of concentrated academic activity. It is partly for this reason that construction and landscape developmental plans for many campuses recognize the need to provide oases for recreation, reflection, and mental restoration. However, there has never been a systematic study of preferences for landscape design relative to the level of restoration experienced after study-induced stress or fatigue. Respondents were recruited from well-visited locations across campus, including library, bookstore, restaurant, and athletic fields. The average amount of time required for completion of the questionnaire was 15 min. The recruitment material was approved by the institutional review board for research on human subjects at the University of California, Irvine.

A cover letter introduced the research project and informed potential respondents that participation is voluntary and confidentiality is assured throughout the entire process. Each survey was denoted by a numerical identifier. Self-reported information was collected on baseline characteristics such as sex, age, level of education attained, marital status, and ethnic background. Information was also collected on the location of permanent domicile and on the length of time that respondents have spent living and working or schooling at the specific campus.

### Topophilia rating.

Restorative environment is used in the context of this study to mean a place associated with relief from mental stress or fatigue. There are few standardized quantitative measures of the specific components of restorative environments (Laumann et al. 2001). In this study, composite measures for environmental perception and preferences for specific ecosystem components and landscape design were integrated in an 18-item questionnaire (questionnaire items 8–25 in Table 1). This measure of topophilia was developed using the theoretical foundations provided by the work of Tuan and existing theories of restorative environments (Betrabet 1996; Hartig and Evans 1993; Hertzog et al. 2003; Tuan 1999). Respondents rated their preferences for specific categories of ecosystem components and environmental and landscape design characteristics on a scale of 1 to 10, with 10 being most effective toward respondent’s expectation of restoration experience. This set of questions focused on the level of importance that respondents accorded to ecosystem components regardless of whether or not they have current access or they expect to actually experience the benefits of exposure to the items being rated. For example, the questions in this category were framed as follows: “Rate the following characteristics (or sensory qualities/ecological components) of an environment according to your expectation of how effective they will be in making you feel refreshed or experience restoration, on a scale of 1–10 with 10 being most effective.”

Confirmatory factor analyses identified four specific domains underlying topophilia: cognitive challenge (e.g., complexity and coherence), synesthetic tendency (e.g., colors and sounds), ecodiversity (e.g., water bodies and trees), and familiarity (e.g., identifiability and privacy). For these domains, statistical factor loadings all exceeded 0.60, and Cronbach α-values ranged from 0.68 to 0.87 (Table 2). The last question in the section on topophilia ratings asked respondents to actually rate the campus according to the number and kinds of accessible restorative environments, on a scale of 1 to 10 with 10 representing saturation (i.e., all subcategories within topophilia are accessible). This question addressed the extent to which various environmental elements were not only present, but also provided satisfying restorative effects in respondents’ current environment. The question was phrased as follows: “On a scale of 1 to 10, rate your current home environment according to the abundance and variety of restorative environments that are accessible to you.”

### Assessing QOL.

I used the brief version of the WHO’s QOL survey instrument (WHOQOL-Bref) in this study to assess the QOL of respondents according to the four minor domains of physical health (seven categorical items), psychological welfare (six items), social relationships (three items), and environmental support (eight items). The four minor domains were statistically modeled to produce an overall score for the QOL for each respondent. The reliability of the associations between the observed variables and the latent domain of QOL was excellent, according to the consistently high Cronbach α-values computed for the models (Table 2). WHOQOL-Bref instrument was used with permission from the WHO (Üstün TB, personal correspondence). A syntax file for checking the data and computing domain scores was obtained from M. Power (University of Edinburgh, Scotland). The WHOQOL-Bref scores were created and interpreted exactly as specified by the WHOQOL Group ( 1994,  1995,  1998a,  1998b). Factor loadings for all four domains exceeded 0.6, and Cronbach α-values ranged from 0.71 to 0.77 (Table 2).

### Statistical analyses.

Descriptive statistics, correlation coefficients, and regression analyses were conducted using SPSS statistical software (version 12.0; SPSS Inc., Chicago, IL). Structural equation modeling to identify relationships among the domains of topophilia and QOL was conducted using Amos software (version 5.0; SPSS, Inc.).

## Results

### Human subjects.

Table 1 shows the descriptive statistics and general properties of the sample population. A total of 379 respondents completed the questionnaire. The average age of respondents was 23 years, ranging from 17 to 60 years. Females represented 58% of the sample population. The sample was ethnically diverse, but of those who registered their ethnicity, more respondents (24%) claimed Asian ethnicity than others (17% Caucasian, 4% Hispanic, 3% African American, and 4% mixed ethnicity). The majority (88%) of the sample population reported being single (9% married; 3% divorced or separated). Most respondents (79%) were pursuing undergraduate degree programs, and a large majority (88%) reported themselves to be healthy at the time of the survey.

### Statistical model.

**Figure 1:**
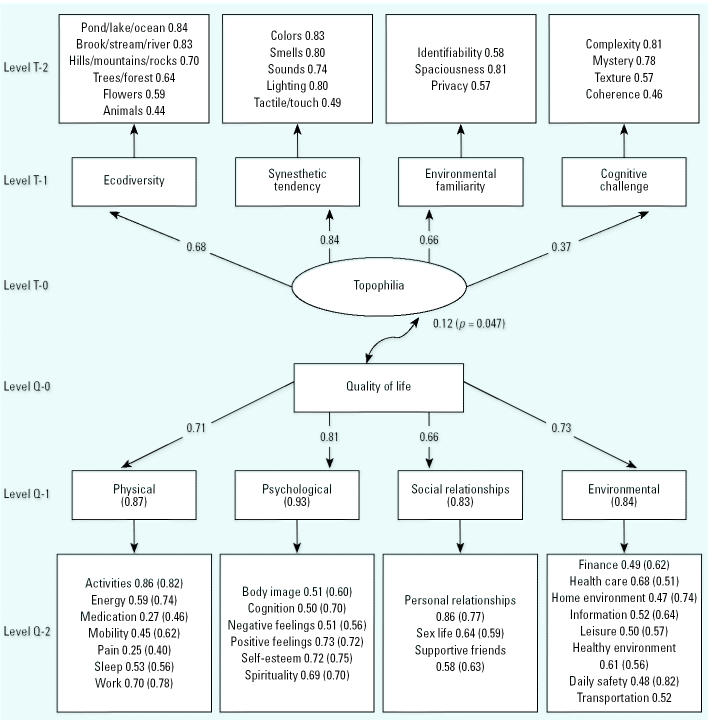
The statistical model of the association between topophilia, QOL, and their proximate determinants. Structural equation modeling was used to generate confirmatory loading factors for the relationships between each of the questionnaire items for topophilia and the standardized WHOQOL-Bref model. Boxes at level T-1 represent the four major domains of topophilia that were revealed by principal components analysis of responses to rating preferences for questionnaire items included in the boxes at level T-2. Level T-0 in the oval shape represents the latent variable of topophilia. Similarly, boxes at level Q-1 represent the four major domains of QOL identified through principal components analysis of responses to questionnaire items at level Q-2. Level Q-0 in the rectangular shape represents measured values for QOL. The factor values not in parentheses are from this study; comparative values for an international field trial of WHOQOL-Bref are included (in parentheses) from the general instrument validation study reported by Skevington et al. (2004).

It was important to first determine whether the responses to questions posed to assess topophilia clustered together in easily recognizable groups. Indeed, confirmatory factor analyses demonstrated four domains underlying topophilia: ecodiversity (questionnaire items 20–25 in Table 1), synesthetic tendency (items 15–19), cognitive challenge (items 8–11), and familiarity (items 12–14). Structural equation modeling showed that all four domains loaded onto the latent construct of topophilia. The strongest domain was synesthetic tendency (0.84), and the weakest domain was cognitive challenge (0.37) (Figure 1).

Four major domains of human experience are also generally recognized to contribute to human self-reporting of QOL. Figure 1 shows the results of confirmatory factor analyses demonstrating that the four recognized domains of WHOQOL-Bref (i.e., physical health, psychological well-being, social relationships, and environmental support) also loaded highly on the underlying latent construct of QOL. These factor loadings are comparable with those identified in an international population sample by the WHOQOL Group ( 1994,  1995,  1998a,  1998b). The strongest domain was psychological well-being (0.81), and the weakest domain was social relationships (0.66).

I also used structural equation modeling to test the relationship between the latent variable of topophilia and the overall QOL scores based on WHOQOL-Bref. The statistical model showed extremely good fit with the data, linking observed overall QOL score and the latent variable of topophilia that was derived from all the four major domains: χ^2^ (df = 5, *n* = 379) = 5.02 (*p* = 0.414). The correlation between topophilia and QOL score is 0.12 (*p* = 0.047) (Figure 1). The smallest loading factor among the four underlying determinants of topophilia was 0.37 for the domain of cognitive challenge. Therefore, I tested a new model without the cognitive challenge domain, and the fit between the data and model improved slightly: χ^2^ (df = 2, *n* = 379) = 1.84 (*p* = 0.398). For this new model, the correlation between topophilia and QOL remained at 0.12 (*p* = 0.040). Therefore, I judged the model with all four domains of topophilia to be the best model, although further research is warranted to improve the factor loading for the cognitive challenge domain, which currently includes questions on complexity, mystery, coherence, and texture.

### Variance and correlations among the domains of topophilia and QOL. Topophilia.

On a scale of 1 to 10, with 10 being the most effective in supporting a restorative experience, the mean rating of topophilia subcategories ranged from the lowest observed value of 4.75 (SD = 2.67) for complexity to the highest observed value of 7.90 (SD = 2.32) for the presence of trees (Table 1). The mean (±SD) rating of restoration opportunities attributed to respondents’ location was 7.1 ± 1.9, also on a scale of 1–10, with 10 being the most saturated with opportunities for experiencing restoration.

### Quality of life.

Most respondents ranked their QOL very highly (mean ~ 3.98 ± 0.81; on a scale of 1–5, with 5 being the highest QOL). Similarly, most respondents were satisfied with their health status (mean = 3.69 ± 0.89). Respondents mostly felt that their lives are meaningful (mean = 3.76 ± 0.96), and most enjoyed a healthy physical environment (mean = 3.54 ± 0.83). The computed scores for the four domains of QOL were reasonably high, consistent with scores observed by WHOQOL Group ( 1994,  1995,  1998a,  1998b) for healthy international populations. The computed score for the physical health domain was the highest (mean = 15.22 ± 2.24; on a scale of 1–20, with 20 being the highest). The lowest domain score was for the environment domain (mean = 14.38 ± 2.33). The overall QOL computed from the domain scores was also high (mean = 14.69 ± 2.11) (Table 1).

### Correlations.

Table 3 shows the correlation matrix between the domains of topophilia and the domains of QOL. The data show that only the “ecodiversity” category of topophilia was significantly correlated to the overall QOL (*r* = 0.123; *p* < 0.05), and within this category, the presence of flowers (*r* = 0.162; *p* < 0.01) and proximity to lakes/ocean (*r* = 0.129; *p* < 0.05) were significantly correlated with the overall QOL. All the major categories of topophilia were significantly correlated with the rating of opportunities for restoration at the current domicile of the respondents, but the domain of familiarity was significant only at the *p* = 0.05 level, whereas cognitive challenge, synesthetic tendency, and ecodiversity were significant at the *p* = 0.01 level (Table 3).

## Discussion

What are the tangible health benefits to its citizens of society’s investment in ecologic conservation, environmental design, and expensive landscape architecture? There is near universal agreement that these investments are justifiable, but until now there have been no straightforward methodologies for providing quantitative answers to this question because of the widely acknowledged variations in individual preferences and valuation of environmental quality across regional, national, political, and cultural boundaries. This study linked, for the first time, a standardized globally validated measure of human QOL with the indicators of human preferences for ecosystem attributes that have been associated with restorative environments. In addition to providing this linkage, the results of this study also suggest a quantitative strategy for proactive assessment of user preferences for specific landscape features before the implementation of environmental design initiatives aimed at enhancing public health and welfare.

This study was conducted primarily among an educated youthful population sample inhabiting a societal microcosm. This is considered an important strength of the study in the sense that both the population and site are supported by considerable societal economic expenditure as an investment in future generations. However, appropriate caution is warranted before the data can be extrapolated to major urban centers—for example, in the construction of large parks for populations having lower levels of education, different ethnic composition, or different kinds of stressors. That said, it is important to note that the WHOQOL-Bref model scores observed in this study are not significantly different from those measured for healthy populations in most parts of the world (Saxena et al. 2001; Skevington et al. 2004) (Figure 1).

This study yielded two major findings: *a*) The overall QOL score is significantly associated with high rating of topophilia, and *b*) environmental and landscape design strategies associated with cognitive challenge—complexity, coherence, and the use of textural stimulation—are less effective in creating impressions of environmental restoration, whereas ecologic designs using ecodiversity themes—particularly the presence of flowers, lakes, or oceans—are generally perceived as providing restorative environments. The implications of these two major findings are discussed in the following sections.

### Linkage of topophilia, restoration, and QOL.

The major finding of this study is that a statistically valid model explicitly connects a standardized measure of the overall QOL scores with the latent construct of topophilia (correlation = 0.12; *p* = 0.047). Furthermore, all the factor loadings from the four precisely defined domains (ecodiversity, synesthetic tendency, environmental familiarity, and cognitive challenge) were significant, and the reliability according to Cronbach α-values was very good for the latent construct of topophilia (Table 2). These findings provide a strong tool for studies attempting to bridge the current epistemologic gap between personal preferences for environmental or ecologic resources and mental health. There is a long history of research on the theoretical underpinnings of the specific identities of person–environment interactions that enhance the restorative experience (Betrabet 1996; Kaplan 1995, 2001; King et al. 2002; Korpela et al. 2001). However, empirical validations of these theoretical constructs are rare. Among the dominant theories of restorative environments is attention restoration theory (ART), which posits that intensive or prolonged use of directed attention leads to fatigue of the mechanisms that serve it, and that the recovery of effective functioning (restoration) is enabled by experience of certain components of a restorative environment (Hertzog et al. 2003; Kaplan 1995). ART is particularly relevant to populations encamped in densely populated geographical locations with the fatigue-prone occupations. According to ART, restorative environments are characterized by four features: “being away,” “extent,” “fascination,” and “compatibility” (Hertzog et al. 2003). The topophilia domains used in the present study differ substantially from ART features, although there are overlaps. For example, certain aspects of “being away” and “compatibility” are captured by the “environmental familiarity” index used in this study. Similarly, the “extent” feature of ART is most similar to the “cognitive challenge” category, whereas the “fascination” feature of ART is most similar to the “synesthetic tendency” construct used here. Perhaps the most salient advantage of the strategy used here is the explicit presentation of “ecodiversity” as a category. In ART, the main focus is to explain why people prefer natural environments to artificial (built) environments. This limitation has prevented empirical analysis of just what part of nature people find extensive, fascinating, or compatible. The finding of the present research eliminates this limitation and provides a solid context for further empirical testing of the determinants of restorative environments.

### Ecodiversity themes are paramount in the environmental restoration experience.

The results of this research further buttress previous findings that when presented with opportunities for restoration, people rank proximity to natural/wildlife environments higher than landscape or urban constructions that overemphasize complex designs or artificial sensory stimulation, although these latter criteria can also contribute to the overall restoration experience. Specifically, the presence of flowers and water bodies are identified in this study as major factors that are associated with QOL and the experience of restorative environments. This level of pinpointing has been previously difficult to establish because most research on environmental preferences have relied on composite measures of “nature,” such as photographs of forests or nature hikes (Hartig et al. 1994). Specifically, van den Berg et al. (2003) noted that the absence of mediational analyses in past research has led to inadequate evidence for the intricacies of the theoretically sound and empirically supported line of reasoning that people typically demonstrate a fondness for nature more than the built environment. The functional accounting of environmental preferences suggests that individuals are attracted to environments that provide tangible benefits to health and that the level of attraction depends on the baseline of measurable health status (Hertzog et al. 2003). To use a pertinent metaphor, drivers whose automobiles rarely run out of gas are also more likely to pay attention to their fuel gauges and to know the locations of the best refueling stations, being picky about the cost of fuel and brand name of each station. That is, they are more likely to indulge in preferential rating of refueling stations than drivers who are stressed and less attentive. To bring this metaphor home to the present study, those who maintain a high QOL are also more likely to rank high on topophilia and to more clearly identify those components of the environment that afford high levels of regular restoration.

In addition to pointing out the positive associations between specific components of ecodiversity and mental health, it is also noteworthy to emphasize the surprising finding that none of the components of the synesthetic sensory stimuli category showed strong statistical association with QOL. So, for example, the anecdotal linkages that have been made in the academic literature, and even in commercial enterprises regarding the health benefits of listening to sounds associated with wildlife and natural settings (e.g., ocean waves, wind-rustled leaves, cricket sounds), are not strongly supported here. However, it is equally important to note the subjective nature of such preferences, and a much larger subject sample may be required to reach firm conclusions in this direction.

## Conclusion

This study demonstrated a statistically significant association between QOL and topophilia using a standardized, internationally validated measure of QOL developed by the mental health group of the WHO, and a new construct of environmental preferences defined by the latent variable of topophilia. Synesthetic tendency is the strongest domain of topophilia, and psychological well-being is the strongest domain of QOL. Furthermore, the study demonstrated in the sample population that the appreciation of ecologic diversity is the strongest component of topophilia that is associated with QOL. Within the ecodiversity subdomain, the appreciation of flowers and water bodies are correlated with high QOL, but not the presence of animals, trees, or hilly terrains. These findings are consistent with other findings regarding the ubiquitous preference of natural environments instead of built environments, in the sense that no strong associations were observed between environmental features of complexity and coherence, which are typically assumed to be artificial features. In addition, there were no strong associations between the experience of sensory stimuli, such as sound, smell, or color, and QOL, possibly because of a high level of variance in the latent variable entitled synesthetic tendency. This study provides a new empirical way of assessing restoration and other health benefits that have been theoretically associated with human experience of specific ecosystem components. The approach presented here should be valuable for proactive environmental and landscape design with the aim of providing mental restoration after stress and fatigue.

## Figures and Tables

**Table 1:** Descriptive statistics of the sample population and the summary statistics of responses to the composite questionnaire (*n* = 379).

Questionnaire item	Minimum	Maximum	Mean ± SD
1 Participant’s sex	0 (Male)	1 (Female)	0.58 ± 0.495
2 Date of birth (year only)	1943	1986	1979 ± 5.477
3 Level of education completed	0	5	2.09 ± 1.488
4 Marital status	0	4	0.19 ± 0.605
5 Ethnicity	1	9	5.46 ± 3.468
6 Level of restoration experienced off campus	1	5	2.56 ± 5.152
7 Level of restoration experienced on campus	1	5	3.11 ± 7.411
8 Environmental complexity rating	1	10	4.75 ± 2.673
9 Environmental mystery rating	1	10	5.04 ± 2.661
10 Environmental coherence rating	1	10	5.97 ± 2.354
11 Environmental texture rating	1	10	5.83 ± 2.442
12 Environmental identifiability rating	1	10	7.29 ± 2.633
13 Spaciousness rating	1	10	7.69 ± 2.555
14 Privacy rating	1	10	7.08 ± 2.496
15 Colors rating	1	10	7.38 ± 2.385
16 Smells rating	1	10	7.24 ± 2.566
17 Sounds rating	1	10	7.35 ± 2.521
18 Light rating	1	10	7.59 ± 2.410
19 Tactile (touch stimulation) rating	1	10	6.06 ± 2.467
20 Flowers rating	1	10	7.27 ± 2.515
21 Trees rating	1	10	7.90 ± 2.324
22 Animals rating	1	10	6.00 ± 2.814
23 Flowing water rating	1	10	7.68 ± 2.605
24 Lake or ocean rating	1	10	7.79 ± 2.538
25 Hills or mountains rating	1	10	7.11 ± 2.642
26 Campus rating on topophilia criteria	1	10	7.08 ± 1.942
27 Currently ill?	1	5	1.02 ± 0.259
28 How do you rate your quality of life?	1	5	3.98 ± 0.814
29 How well are you satisfied with your health?	1	5	3.69 ± 0.893
30 What extent does physical pain hamper you?	1	5	4.13 ± 0.999
31 Need medical treatment to function?	1	5	4.38 ± 0.893
32 Enjoy life?	1	5	3.88 ± 0.826
33 Feel life to be meaningful?	1	5	3.76 ± 0.956
34 Able to concentrate?	1	5	3.31 ± 0.883
35 Safe in daily life?	1	5	3.86 ± 0.787
36 Healthy physical environment?	1	5	3.54 ± 0.830
37 Enough energy for daily life?	1	5	3.73 ± 0.808
38 Accept your bodily appearance?	1	5	3.57 ± 0.976
39 Enough money to meet your needs?	1	5	3.32 ± 1.205
40 Information that you need available?	1	5	3.71 ± 0.775
41 Opportunity for leisure activities?	1	5	3.27 ± 0.964
42 Able to get around?	1	5	3.91 ± 0.935
43 Satisfied with your sleep?	1	5	3.27 ± 1.048
44 Satisfied with ability for daily activities?	1	5	3.68 ± 0.826
45 Satisfied with capacity for work?	1	5	3.55 ± 0.922
46 Satisfied with yourself?	1	5	3.78 ± 0.916
47 Satisfied with your personal relationships?	1	5	3.68 ± 1.038
48 Satisfied with your sex life?	1	5	3.31 ± 1.186
49 Satisfied with support from friends?	1	5	3.91 ± 0.915
50 Satisfied with conditions of living space?	1	5	3.71 ± 0.961
51 Satisfied with access to health care?	1	5	3.68 ± 0.982
52 Satisfied with transport?	1	5	3.65 ± 1.131
53 How often do you have negative feelings?	1	5	3.59 ± 0.864
54 Physical domain	6.86	20.00	15.2153 ± 2.24354
55 Psychological domain	6.67	20.00	14.5933 ± 2.49269
56 Social domain	4.00	20.00	14.6029 ± 3.38493
57 Environment domain	5.00	20.00	14.3765 ± 2.33073
58 Overall QOL score	7.18	19.58	14.6985 ± 2.11191
59 Ecodiversity ratings factor	1.00	10.00	7.3687 ± 1.94399
60 Synesthetic tendency ratings factor	1.00	10.00	7.3917 ± 2.09584
61 Cognitive ratings factor	1.00	10.00	5.5342 ± 1.79975
62 Familiarity ratings factor	1.00	10.00	7.3611 ± 2.01276

**Table 2:** Cronbach α-value estimates of statistical reliability for the associations between observed variables (minor domains) and the two latent variables of topophilia and QOL (major domains).

Domains	α-Value
Topophilia	
Ecodiversity	0.833
Synesthetic tendency	0.870
Cognitive challenge	0.746
Familiarity	0.684
QOL	
Physical health	0.717
Psychological well-being	0.777
Social relationships	0.715
Environmental support	0.751

**Table 3:** Matrix of correlation coefficients among QOL, topophilia, and respondent experience of restoration.

	QOL domains					
Topophilia domains	Physical	Psychological	Social	Environmental	Overall QOL	Level of restoration at current location
Cognitive					(0.009)	
Complexity	−0.004	0.029	0.043	−0.030	0.017	0.189**
Mystery	−0.019	−0.002	0.095	−0.056	0.016	
Coherence	−0.016	−0.062	0.003	−0.005	−0.024	
Texture	−0.061	−0.078	0.047	−0.029	−0.031	
Familiarity					(0.082)	
Identifiability	0.041	0.006	0.072	0.115*	0.069	0.118*
Spaciousness	0.049	0.014	0.095	0.009	0.054	
Privacy	0.034	0.092	0.067	−0.006	0.062	
Synesthetic tendency					(0.077)	
Colors	0.036	0.049	0.059	0.041	0.059	0.183**
Smells	−0.013	0.010	0.084	−0.005	0.030	
Sounds	0.062	0.081	0.058	0.030	0.071	
Lighting	0.083	0.092	0.062	0.072	0.095	
Tactile	−0.002	−0.005	0.084	−0.010	0.033	
Ecodiversity					(0.123*)	
Flowers	0.128*	0.063	0.188*	0.106*	0.162**	0.282**
Trees	0.087	0.012	0.082	0.073	0.084	
Animals	−0.023	−0.033	0.063	0.024	0.021	
Flowing water	0.053	−0.005	0.055	0.059	0.055	
Lake/ocean	0.082	0.064	0.136*	0.105*	0.129*	
Hills/mountain	0.040	0.039	0.075	0.067	0.075	

Values in parentheses are correlation coefficients between the overall QOL and each of the major domains of topophilia tested as a group.

*Pearson correlation coefficients are significant at the 0.05 level (two-tailed).

**Pearson correlation coefficients are significant at the 0.01 level (two-tailed).
